# Protective Effects of 2-Amino-5,6-dihydro-4*H*-1,3-thiazine and Its Derivative against Radiation-Induced Hematopoietic and Intestinal Injury in Mice

**DOI:** 10.3390/ijms19051530

**Published:** 2018-05-21

**Authors:** Yuanyuan Li, Shaofan Kong, Fujun Yang, Wenqing Xu

**Affiliations:** Institute of Radiation Medicine, Chinese Academy of Medical Science and Peking Union Medical Collage, Tianjin Key Laboratory of Radiation Medicine and Molecular Nuclear Medicine, Tianjin 300192, China; yuanyuanli0929@163.com (Y.L.); ksf226@126.com (S.K.); yangfj32@126.com (F.Y.)

**Keywords:** 2-amino-5,6-dihydro-4*H*-1,3-thiazine hydrobromide (2-ADT), 2-acetylamino-5,6-dihydro-4*H*-1,3-thiazine hydrobromide (2-AADT), radioprotection, hematopoietic, intestinal injury

## Abstract

Ionizing radiation (IR) acts as an external stimulating factor, when it acts on the body, it will activate NF-κB and cause the up-regulation of inducible nitric oxide synthase (iNOS) and induce a large amount of nitric oxide (NO) production. NO and other reactive nitrogen and oxygen species (RNS and ROS) can cause damage to biological molecules and affect their physiological functions. Our study investigated the protective role of 2-amino-5,6-dihydro-4*H*-1,3-thiazine hydrobromide (2-ADT) and 2-acetylamino-5,6-dihydro-4*H*-1,3-thiazine hydrobromide (2-AADT), two nitric oxide synthase inhibitors, against radiation-induced hematopoietic and intestinal injury in mice. Pretreatment with 2-ADT and 2-AADT improved the survival of mice exposed to a lethal dose of radiation, especially, the survival rate of the 2-ADT 20 mg/kg group was significantly higher than that of the vehicle group (*p* < 0.001). Our findings indicated that the radioprotective actions of 2-ADT and 2-AADT are achieved via accelerating hematopoietic system recovery, decreasing oxidative and nitrosative stress by enhancing the antioxidant defense system and reducing NO as well as peroxynitrite (ONOO${}^{-}$) content, and mitigating the radiation-induced DNA damage evaluated by comet assay. These results suggest that 2-ADT and 2-AADT may have great application potential in ameliorating the damages of radiotherapy.

## 1. Introduction

Radiotherapy is an important treatment modality for various malignancies and severe diseases [1], but these adverse side effects such as hematopoietic and gastrointestinal dysfunction even death caused by radiation reduce the patients’ quality of life and limit their radiation therapy. Ionizing radiation-induced injury is mediated either directly or indirectly through the radiolysis of water and production of reactive oxygen species (ROS), including hydroxyl radical (·OH), O${}_{2}$${}^{-}$ and H${}_{2}$O${}_{2}$ [2]. Furthermore, gamma-irradiation can enhance endogenous NO biosynthesis by iNOS from L-arginine in vivo [3,4]. NO can react rapidly with O${}_{2}$${}^{-}$ to form ONOO${}^{-}$, a potent cytotoxic oxidant. ROS as well as ONOO${}^{-}$ and other reactive nitrogen species (RNS) can attack virtually all components including DNA, lipids and proteins, resulting in impaired cellular functions and enhanced inflammatory responses [5,6,7]. Previous studies have proposed that ROS are the initiators and that NO and other RNS are the effectors activating cytosolic signal transduction pathways in response to ionizing radiation [4,8]. It is therefore increasingly necessary to develop effective compounds to protect the patients from radiation-induced injury.

For a long period of time, radioprotective agents have been mostly concentrated on antioxidants that are targeted for the scavenging of ROS, but less attention was given to compounds that alleviate the nitrosative stress caused by nitric oxide and its derivatives. Published studies have shown that ionizing radiation induces the expression and binding activity of NF-κB [9], and high-dose ionizing radiation causes activation of NF-κB in different tissues (such as spleen and bone marrow) in vivo [10]. Moreover, NF-κB is involved in the induction of iNOS gene [11], which increases the production of NO [6]. Therefore, NO scavenger or iNOS inhibitor may weaken the nitrosative stress after irradiation by reducing the content of NO in the tissues, thus exerting radiation protection. Many facts confirm the practical significance of chemical agents in achieving radioprotection under the NO-dependent mechanism. It has been reported that the radiation protection of NOS inhibitors afforded by manipulations of NO levels was due to bone marrow protection, which was mediated by changes in blood flow and oxygen delivery [12]. Suppression of NOS activity in vivo by NOS inhibitors results in an increase in the number of stem and early progenitor cells in the bone marrow and of neutrophils in the blood after irradiation [2,13]. Evidence that the leading mechanism of the radioprotective effect of NOS inhibitors among isothiourea derivatives is the induction of hypoxia was provided by Filimonova MV in 2014 [14]. Drugs such as catecholamines, serotonin and other vasoactive agents also provide radiation protection by inducing tissue hypoxia through their pharmacological activities in bone marrow or spleen [15,16,17]. the above studies have prompted us to consider chemical NOS inhibitors as possible directions for finding new radioprotector.

2-amino-5,6-dihydro-4*H*-1,3-thiazine hydrobromide (2-ADT) and 2-acetylamino-5,6-dihydro- linebreak 4*H*-1,3-thiazine hydrobromide (2-AADT), which are potent inhibitors of mammalian NO synthases, exhibited vasopressor action in experimental models of rat endotoxic shock [18,19,20]. It is known that the primary cause of death from radiation sickess is hematopoietic and gastrointestinal syndromes associated with sepsis [2]. Here, we investigated the protective effect of 2-ADT and 2-AADT on hematopoietic and intestinal damage in irradiated mice. In this study, the 30-day survival rate of mice, effects of 2-ADT and 2-AADT on hematopoietic system, antioxidant defense system, DNA damage, NO content, the crypt-villus structure of the small intestine and proliferation and differentiation of intestinal cells in irradiated mice were respectively assessed.

## 2. Results

### 2.1. 2-ADT and 2-AADT Improved the Survival of Mice Exposed to a Lethal Dose of Irradiation

**Figure 1:**
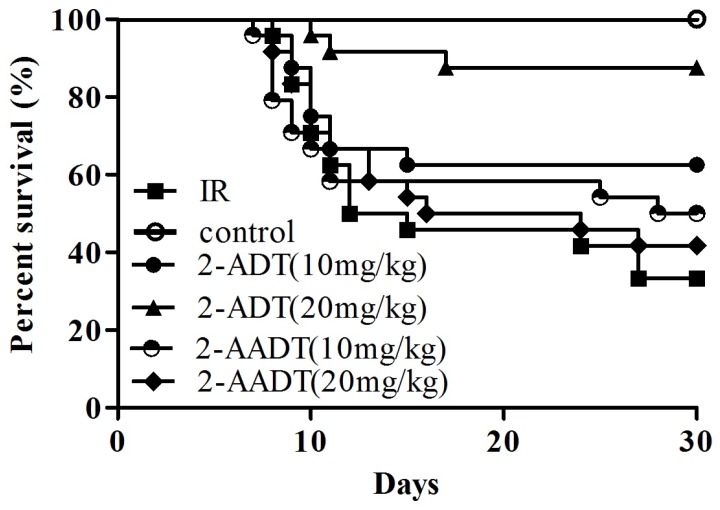
Survival curve of mice exposed to 7.5 Gy WBI. Mice (*n* = 24 each) were injected i.p. with 2-ADT or 2-AADT 30 min before being exposed to 7.5 Gy WBI. Comparison of survival curves by Kaplan-Meier survival analysis and the log-rank (Mantel-Cox) test. the difference in survival between the vehicle+IR and 2-ADT 20 mg/kg group was statistically significant (*p* < 0.001).

As shown in Figure 1, Only 33.3% (8/24) of the vehicle-treated group survived for 30 days following 7.5 Gy whole body irradiation. the survival rates of 2-ADT (10 mg/kg, 20 mg/kg) and 2-AADT (10 mg/kg, 20 mg/kg) groups increased by 29.2%, 54.2%, 16.7% and 8.3%, respectively, compared to the vehicle-treated group. Moreover, the survival rate of 2-ADT 20 mg/kg group was significantly higher than that of vehicle group (*p* < 0.001).

### 2.2. 2-ADT and 2-AADT Promoted Recovery of Hematopoietic System

**Figure 2:**
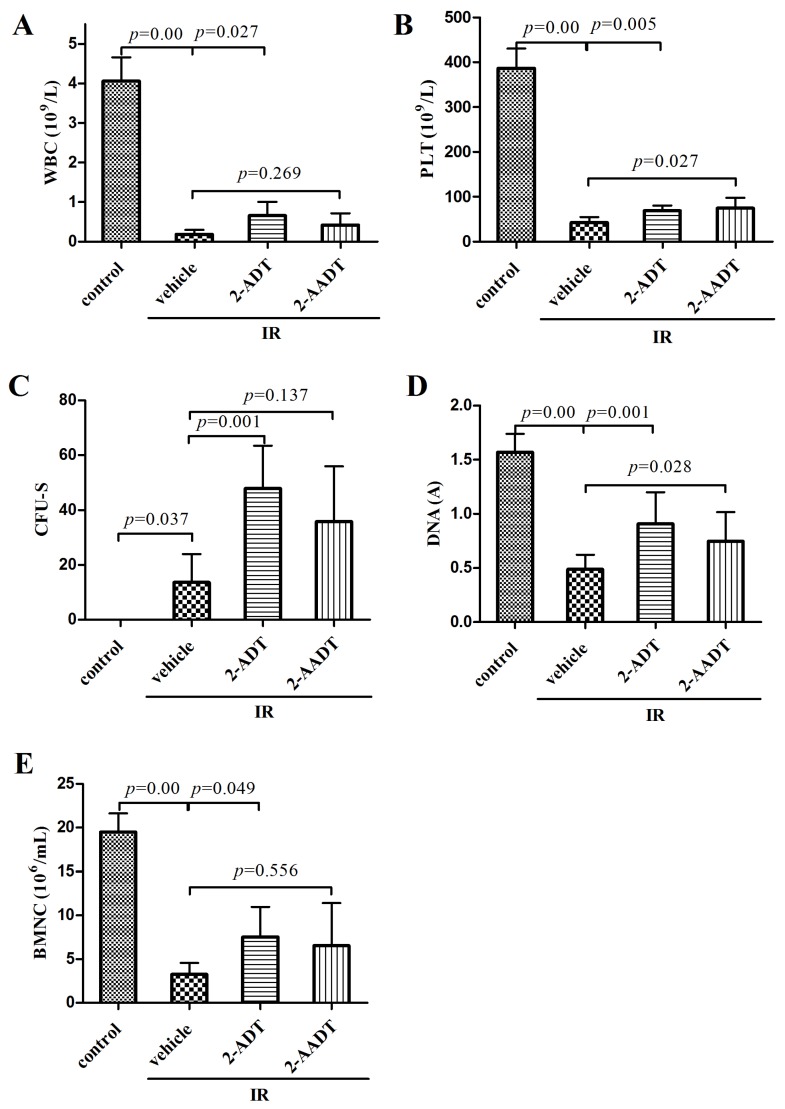
Effects of 2-ADT and 2-AADT on hematopoietic system. (**A**) WBC counts; (**B**) PLT counts; (**C**) CFU-S counts; (**D**) DNA content and (**E**) BMNC counts of irradiated and control mice 7 days after treatment. 2-ADT and 2-AADT were administered i.p. 30 min before 6.0 Gy irradiation. Results are expressed as the mean ± SD (*n* = 8 each). One-way ANOVA was used for statistical comparisons between groups. the *p* value indicates the difference compared with the IR + vehicle group.

As shown in Figure 2A,B, compared with control group, the numbers of white blood cell (WBC) and platelet (PLT) in irradiated vehicle group were significantly reduced (*p* < 0.01). the 2-ADT treatment group showed markedly higher WBC count than vehicle group (*p* < 0.05). the treatment groups receiving 2-ADT and 2-AADT exhibited obvious increase in PLT level compared with vehicle group (*p* < 0.05).

The spleen is an important representative of the hematopoietic organs of mice [15], and the number of endogenous CFU-S is considered to be an index of hematopoietic function [21]. Figure 2C shows that the number of CFU-S in 2-ADT treated group was significantly higher than in vehicle group (*p* < 0.01).

The bone marrow DNA content and the number of BMNC were determined to evaluate the damage and repair of bone marrow cells (Figure 2D,E). Ionizing radiation significantly reduced the bone marrow DNA content in vehicle group compared to the control group (*p* < 0.01), and the DNA content of mice treated with 2-ADT and 2-AADT was significantly higher than that of vehicle group mice (*p* < 0.05). the statistically significant decrease in BMNC counts was observed from the control group to the irradiation vehicle group (*p* < 0.01). Administration of 2-ADT prior to irradiation effectively elevated the BMNC counts (*p* < 0.05).

### 2.3. 2-ADT and 2-AADT Enhanced the Activity of Various Antioxidant Enzymes and Reduced Lipid Peroxidation

**Figure 3:**
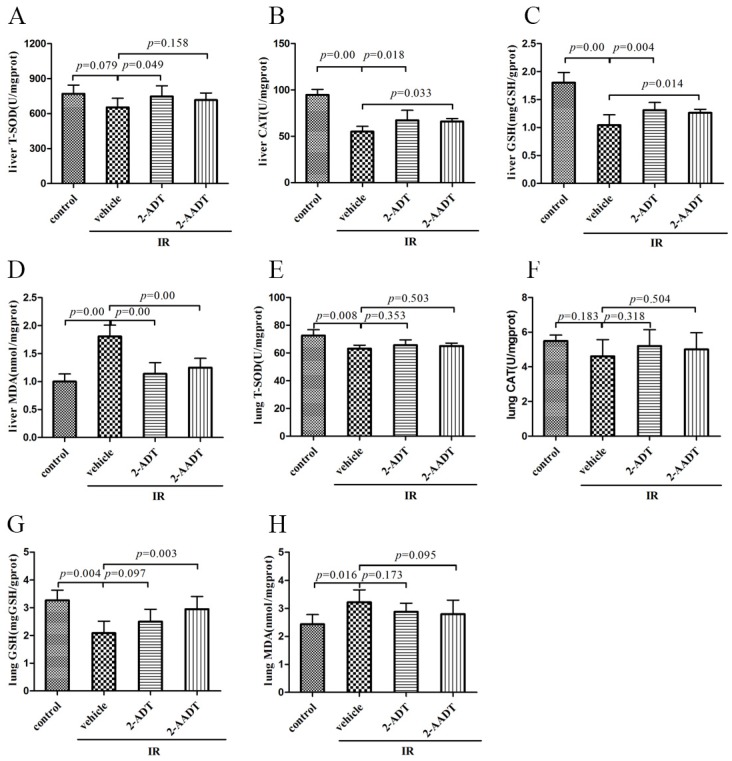
Effects of pretreatment with 2-ADT and 2-AADT on alterations of antioxidant enzymes activities in tissues after irradiation. 2-ADT and 2-AADT were administered i.p. 30 min before irradiation. (**A**) T-SOD; (**B**) CAT; (**C**) GSH and (**D**) MDA from liver 7 days after 6.0 Gy irradiation; (**E**) T-SOD; (**F**) CAT; (**G**) GSH and (**H**) MDA from lung 7 days after 6.0 Gy irradiation. The data are presented as the mean ± SD (*n*= 8 each). One-way ANOVA was used for statistical comparisons between groups. The *p* value shows the difference compared with the IR + vehicle group.

Endogenous antioxidant defense systems include enzymatic antioxidants such as superoxide dismutase (SOD) , catalase (CAT) and glutathione peroxidase (GPx) and nonenzymatic antioxidants such as reduced glutathione (GSH), which can scavenge and reduce a wide range of oxidants [22]. Figure 3A,E show SOD activity in liver and lung. Figure 3B,F show CAT activity in liver and lung. the activity of SOD and CAT in liver and lung lowered in irradiation vehicle group compared with control group, treatment with 2-ADT and 2-AADT can reverse the reduction of SOD and CAT activity to varying degrees. In particular, the 2-ADT and 2-AADT pretreatment groups significantly increased CAT activity in the liver compared to the irradiated vehicle group (*p* < 0.05).

### 2.4. 2-ADT and 2-AADT Attenuated Radiation-Induced Elevation of NO in Plasma

**Figure 4:**
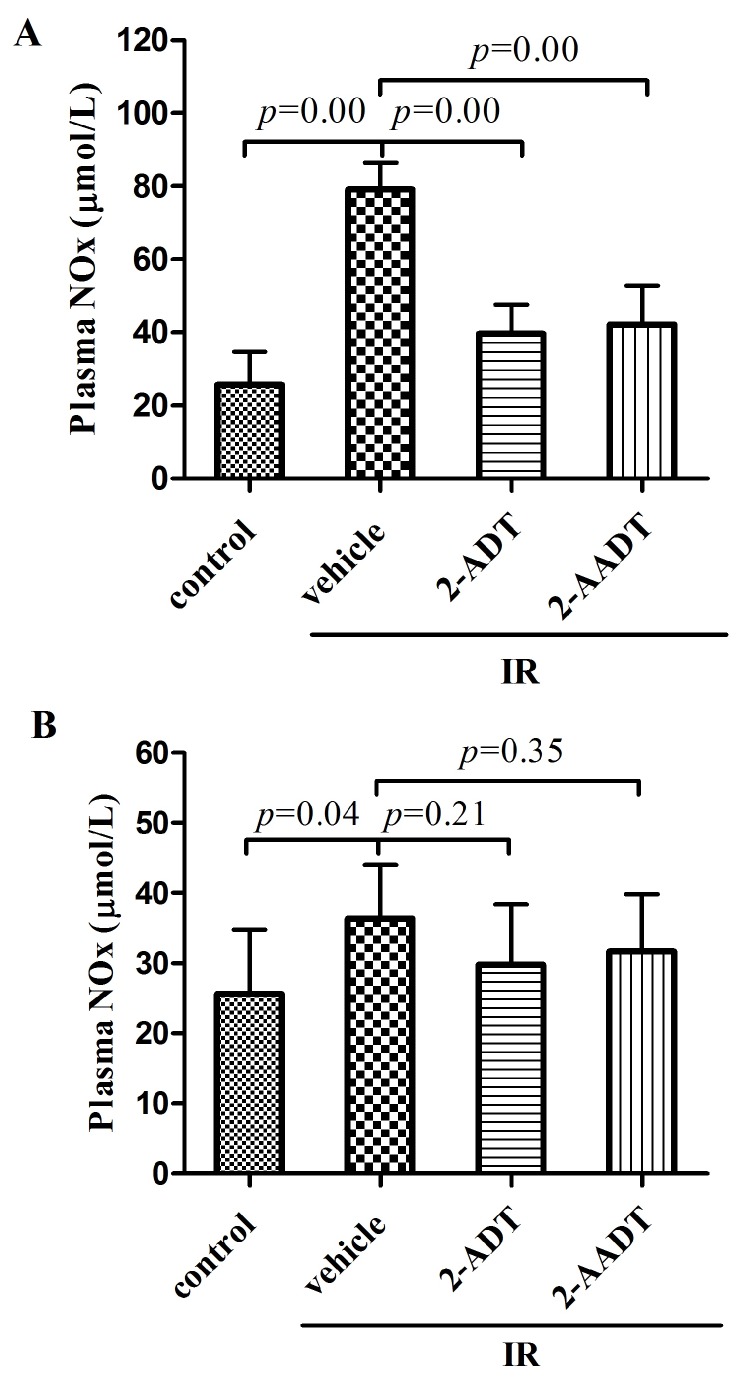
The content of nitrite and nitrate (NOx) in plasma of irradiated and control mice at 6 h and 7 days after 7.5 Gy irradiation. 2-ADT and 2-AADT were administered i.p. 30 min before irradiation. (**A**,**B**) Plasma NOx content at 6 h and 7 days after irradiation, respectively. the data are presented as the mean ± SD (*n* = 12 each). One-way ANOVA was used for statistical comparisons between groups. The *p* value indicates the difference compared with the IR + vehicle group.

Figure 4A,B show the NOx content in plasma, as an index of nitrosative stress [23]. The NO levels (NOx) in plasma were significantly higher in mice subjected to 7.5 Gy WBI at 6 h (*p* < 0.01) and 7 days (*p* < 0.05) than in control mice, but the content at 7 days is much lower than that at 6 h. It is speculated that the content may increase first and then decrease, which is basically consistent with the literature reports [4]. Pretreatment with 2-ADT and 2-AADT significantly (*p* < 0.01) decreased radiation-induced NO production 6 h after irradiation. In contrast, although the 2-ADT and 2-ADDT groups were able to reduce radiation-induced increases in NO levels 7 d after irradiation, they were not significant.

Figure 3C,G show GSH levels in liver and lung. The GSH levels in liver and lung were significantly reduced in mice subjected to 6.0 Gy WBI compared with control mice (*p* < 0.01). Treatment with 2-ADT and 2-AADT resulted in evident increase in GSH content in the liver compared to vehicle group (*p* < 0.01 and *p* < 0.05 respectively). A marked increase of GSH content in the lung was observed only in the 2-AADT treatment group compared to the vehicle group (*p* < 0.01)

Figure 3D,H show malondialdehyde (MDA) levels in liver and lung, as an indicator of lipid peroxidation and oxidative stress [24]. Compared with the control group, the levels of MDA in the liver and lung were significantly elevated in the irradiation vehicle group (*p* < 0.05). Both 2-ADT and 2-AADT sharply decreased liver MDA levels in both treatment groups (*p* < 0.01). However, treatment with 2-ADT and 2-AADT resulted in no significant decrease in lung MDA levels compared to vehicle group (*p* > 0.05).

### 2.5. 2-ADT and 2-AADT Alleviated Radiation-Induced DNA Damage to Peripheral Blood Lymphocytes

**Figure 5:**
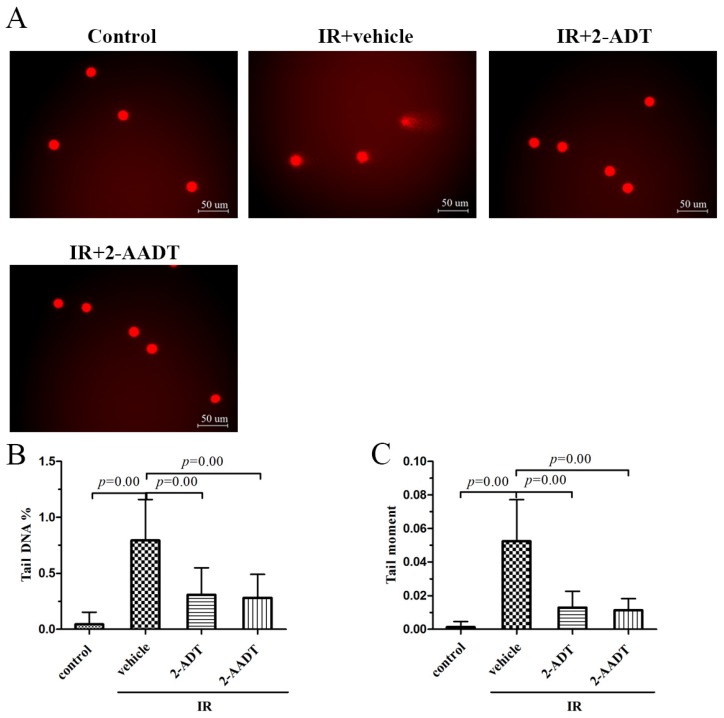
The effects of 2-ADT and 2-AADT on DNA damage in mice after 6.0 Gy radiation. 2-ADT and 2-AADT were administered i.p. 30 min before irradiation. (**A**) Representative images of lymphocytes from different groups. Scale bar, 50 μm; (**B**) Tail DNA% and (**C**) Tail Moment (TM) reflecting DNA damage indirectly by the column graph. the data are presented as the mean ± SD. One-way ANOVA was used for statistical comparisons between groups. The *p* value shows the difference compared with the IR + vehicle group.

The comet assay was used to detect radiation-induced DNA damage, particularly DNA strand breaks, and the percentage of DNA in tail (Tail DNA %) and tail moment (TM) were used as parameters to measure DNA damage [25]. As presented in Figure 5, compared with control group, the Tail DNA% and TM of irradiated vehicle group increased significantly (*p* < 0.01). Treatment with 2-ADT and 2-AADT resulted in a significant reduction in Tail DNA% and TM (*p* < 0.01) compared to the vehicle group. These data indicated that 2-ADT and 2-AADT effectively attenuated radiation-induced DNA damage in lymphocytes.

### 2.6. 2-ADT and 2-AADT Ameliorated Small Intestinal Injury in Mice after Abdominal Irradiation

**Figure 6:**
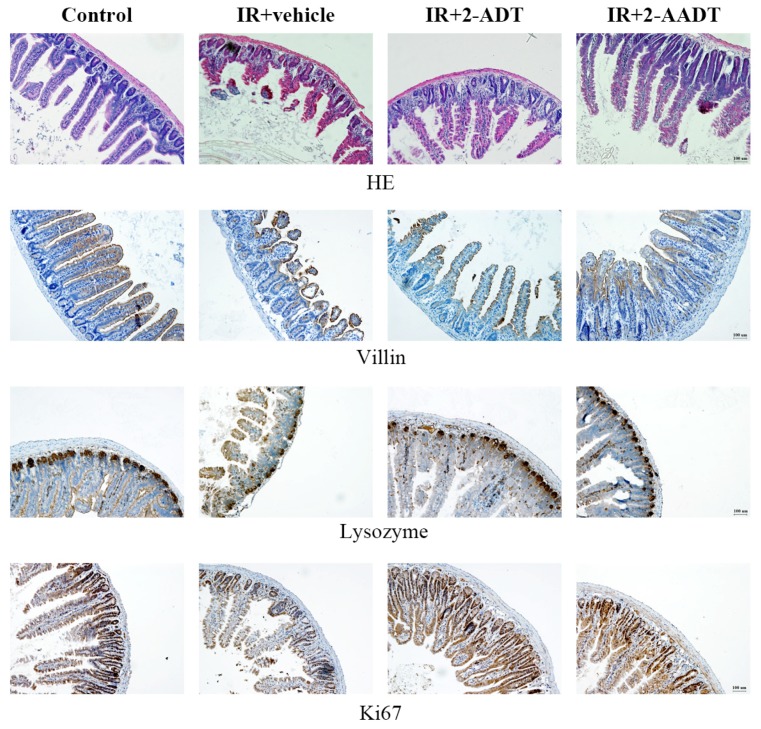
Effects of 2-ADT and 2-AADT on intestinal injury in mice after 13 Gy abdominal irradiation. 2-ADT and 2-AADT were administered i.p. 30 min before irradiation.the small intestine of mice was harvested 3 days after abdominal irradiation for pathological and immunohistochemical examination. Representative HE staining and imunohistochemical images of villin, lysozyme and Ki67 of the small intestine in different treatment groups. Scale bar, 100 μm.

As shown in Figure 6, the architecture of crypt-villus was intact and normal in control group. At 3 days after 13 Gy abdominal irradiation, atrophic and distorted villi , crypt abscesses and loss were observed in the irradiated vehicle group. 2-ADT and 2-AADT treated mice showed increased villi length, more surviving crypts and less crypt abscess than vehicle mice. Thus, pretreatment with 2-ADT and 2-AADT prior to irradiation ameliorated radiation-induced small intestinal injury.

### 2.7. 2-ADT and 2-AADT Promoted the Proliferation of Vill${}^{+}$ Enterocytes, Paneth Cells and Ki67${}^{+}$ Transient Amplifying Cells in the Small Intestine of Mice after Abdominal Irradiation

Immunohistochemical localization of villin, lysozyme and Ki67 antibodies were used to assess the expression of vill${}^{+}$ enterocytes, Paneth cells and Ki67${}^{+}$ transient amplifying cells in the small intestine, respectively [26]. As shown in Figure 6, at 3 days after 13 Gy abdominal irradiation, the numbers of Paneth cells, vill${}^{+}$ and Ki67${}^{+}$ positive cells in vehicle group were significantly lower than that in control group. Pretreatment with 2-ADT or 2-AADT increased the immunostaining of villin, lysozyme and Ki67 compared to the vehicle group. Therefore, 2-ADT and 2-AADT promoted the proliferation and restoration of intestinal injured cells in irradiated mice.

### 2.8. 2-ADT and 2-AADT Attenuated Radiation-Induced Nitrotyrosine Expression in the Small Intestine after Abdominal Irradiation

**Figure 7:**
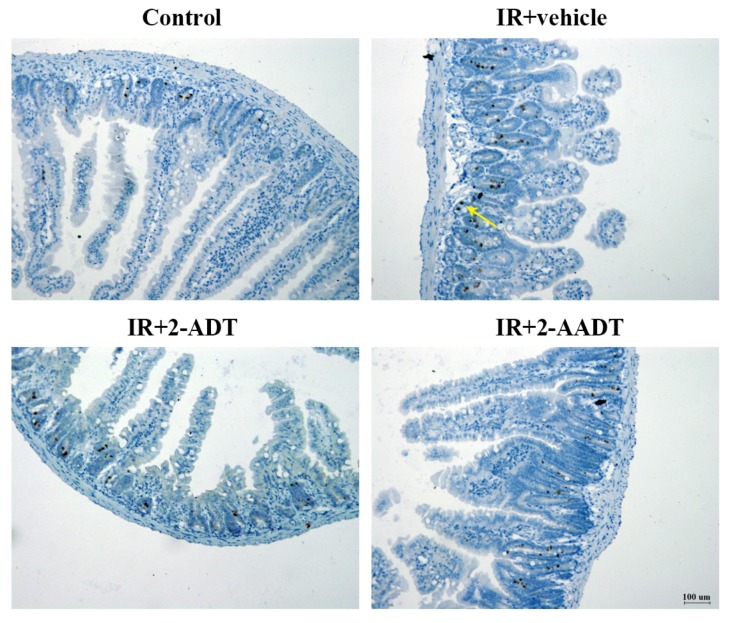
Representative immunohistochemical images of nitrotyrosine in different groups of small intestine on day 3 after exposure to 13 Gy of abdominal irradiation. 2-ADT and 2-AADT were administered i.p. 30 min before irradiation. the yellow arrow indicates nitrotyrosine staining. Scale bar, 100 μm.

Immunohistochemical result of nitrotyrosine was shown in Figure 7, the nitrotyrosine staining in irradiated vehicle group was more intense compared to the weak staining in control group. Mild nitrotyrosine staining was shown in the 2-ADT or 2-AADT treatment group. These results indicate that 2-ADT and 2-AADT can attenuate nitrotyrosine expression in the small intestines.

## 3. Discussion

It is well established that radiotherapy can cause severe toxicity to the organism, mainly due to the interaction of reactive oxygen and reactive nitrogen species with biomolecules such as DNA, lipids and proteins [5]. Furthermore, radiation sickness with basic lesions such as myelosuppression, hematopoietic dysfunction, and gastrointestinal damage has become an important factor restricting radiotherapy [2]. Therefore, there is an urgent need to develop effective drugs to protect normal tissues from radiation damage and improve the prognosis during radiotherapy. A considerable amount of literatures have shown that ionizing radiation can cause a large increase in NO levels, and its excessive production can lead to cytotoxic actions and mediate inflammation [27], and NO is also a mediator of radiation-induced tissue damage and a negative regulator of hematopoietic stem cell activity [7,13]. Evidence suggests that NOS inhibitors can act as radioprotectors by attenuating radiation-induced elevation of NO or inducing hypoxia [2,12,14]. 2-ADT and 2-AADT are potent inhibitors of nitric oxide synthase and early study indicated that the radioprotective activity of 2-ADT was not attributable to the oxygen effect [28]. It is known that 2-AADT is a prodrug of 2-ADT, and 2-AADT has higher NOS inhibitory activity and longer duration of antihypertensive action than 2-ADT [19,20]. However, our study did not find that 2-AADT is significantly more protective than 2-ADT. Because the time of administration is the same, it is likely that the best time for 2-AADT administration is missed. In the endotoxin shock model, the study time is quite short (up to 2 h), but the damage response after radiation is a long-lasting process. Therefore, short-term NOS inhibitory activity does not fully represent its radiation protection. However, inhibitors with strong NOS-inhibiting activity may be candidates for screening radioprotector. the aim of the this study was to evaluate the protective effects of 2-ADT and its derivative 2-AADT on radiation and to explore the underlying mechanism.

In the present study, it was found that both 2-ADT and 2-AADT can prolong the survival of mice exposed to lethal doses, suggesting that they have overall protective effects on the irradiated mice. It is well known that the hematopoietic system is extremely sensitive to IR. Our findings indicated that administration of 2-ADT and 2-AADT increased the amount of endogenous CFU-S, BMNCs, WBC and PLT as well as DNA content, indicating that they promoted the restore and regeneration of the hematopoietic system. In addition, they enhanced the antioxidant defense system of the liver and lungs by potentiating antioxidant enzyme activity, increasing GSH levels and attenuating oxidative stress (Figure 3). Previous studies demonstrated that ROS are the initial reactants generated from irradiation, and RNS is the actual effector of redox-dependent cell signaling pathways [8]. Oxidative stress is closely related to nitrosative stress and RNS. Thus, NOS inhibitors may directly attenuate nitrosative stress by reducing RNS content and indirectly weaken oxidative stress and enhance antioxidant defenses.

It is reported that cytotoxicity, carcinogenicity and mutagenicity in cells exposed to ionizing radiation are thought to be mainly due to damage to DNA [29]. Comet assay results indicated that administration of 2-ADT or 2-AADT can protect DNA from radiation-induced damage. According to previous studies, NO inhibits enzymes involved in mitochondrial respiration, leading to the depletion of intracellular ATP and ultimately to death, and damages DNA directly and indirectly [7,30]. In this study, we found that both of 2-ADT and 2-AADT can reduce the increase of NOx content caused by ionizing radiation in plasma , thereby attenuating NO-mediated cytotoxicity.

In histopathological evaluation of the small intestine based on HE staining, severe damage to crypts and villis was clearly observed, and the histopathological changes considerably reduced in both treatment groups Figure 6. the ONOO${}^{-}$ produced by the rapid reaction of NO and superoxide generated by radiation can lead to membrane lipid peroxidation and nitration and hydroxylation of phenolic compounds (such as tyrosine residues in proteins) [7,31]. Nitrotyrosine, a stable byproduct of ONOO${}^{-}$ as a nitrating agent, is a marker for the presence of peroxynitrite and oxidative damage [7,32]. We detected a significant increase in nitrotyrosine immunostaining in the small intestine on the third day after abdominal irradiation, and we observed that the expression of lysozyme (marker of Paneth cells), villin and Ki67 in 2-ADT and 2-AADT treated group was significant higher than that of the irradiated vehicle group (Figure 6 and Figure 7). It can be deduced that the increase of Paneth cells, vill${}^{+}$ enterocytes and Ki67 cells when 2-ADT and 2-AADT were administered to irradiated mice may be related to suppression of peroxynitrite formation. Study have shown that NO may, via peroxynitrite, alter the proliferative signaling mediated by protein tyrosine kinases, resulting in decreased enterocyte proliferation and differentiation , which together with NO-induced apoptosis leads to intestinal barrier failure [33,34]. Thus, 2-ADT and 2-AADT promote the proliferation of Paneth cells, vill${}^{+}$ enterocytes and Ki67${}^{+}$ cells in the small intestine by attenuating peroxynitrite-mediated nitrosative stress.

In short, the results of this study demonstrated that 2-ADT and 2-AADT as NOS inhibitors have protective effects on radiation-induced hematopoietic and intestinal injury. Our data indicated that the effects were achieved through accelerating hematopoietic regeneration, decreasing oxidative and nitrosative stress by enhancing the antioxidant defense system and reducing NO as well as ONOO${}^{-}$ content, and mitigating the radiation-induced DNA damage. These findings further suggest that 2-ADT or 2-AADT may be effective agents to ameliorate the damages in radiotherapy.

## 4. Materials and Methods

### 4.1. Chemicals

**Figure 8:**
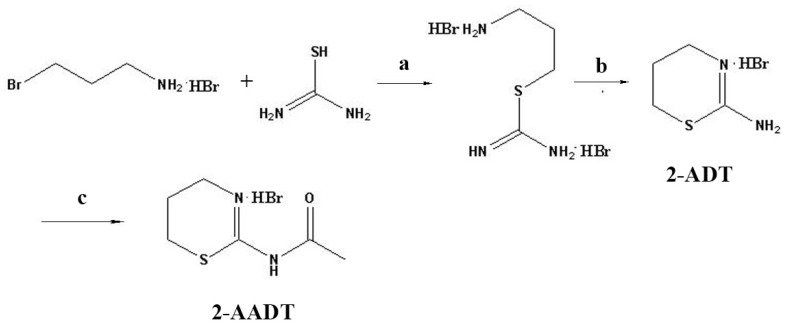
Reagents and conditions: (a) (CH${}_{3}$)${}_{2}$CHOH, 82 ${}^{\circ }$C, 1.5 h; (b) H${}_{2}$O, 100 ${}^{\circ }$C, 21 h; (c) (CH${}_{3}$CO)${}_{2}$O, 90 ${}^{\circ }$C, 10.5 h.

Target compounds 2-ADT and 2-AADT were synthesized as shown in Figure 8. As previously reported in the literature [20], to 1.52 g (20 mmol) of thiourea in 10 mL of isopropanol was added 3-bromopropylamine hydrobromide (5.25 g, 24 mmol), the mixture was refluxed for 1.5 h at 82 ${}^{\circ }$C then cooled. the precipitate was filtered and washed with warm isopropanol, and removal of the solvent under reduced pressure produced a white solid. Yield 4.60 g (78%) of *S*-(aminopropyl)isothiourea dihydrobromide. the obtained compound (4.28 g, 20 mmol) was dissolved in 60 mL of water and was refluxed for 21 h at 100 ${}^{\circ }$C. Then the water was evaporated using rotary evaporator and the rest was twice treated with a hot mixture of isopropanol-ethylacetate (100 mL each). the solutions were combined, evaporated using rotary evaporator till the volume 60 mL and cooled. the precipitate was filtered and washed with ether. Removal of the solvent under reduced pressure yielded an white crystal. Yield 0.85 g (22%) of 2-ADT.

The mixture of acetic anhydride (5 mL) and 2-ADT (0.56 g, 2.8 mmol) was heated at 90 ${}^{\circ }$C under magnetic stirring for 10.5 h and then cooled. the precipitate was filtered off and washed with ether, yielding a pale-yellow powder of 2-ADDT (0.4 g, 60%).

### 4.2. Mice and Irradiation

Male ICR mice (Beijing Vital River Laboratory Animal Technology Co., Ltd., Beijing, China) weighing 20–22 g and male C57BL/6 mice (Beijing HFK Bioscience Co., Ltd., Beijing, China) weighing 20–22 g were used for these experiments. All mice were housed in a climate-controlled, specific pathogen free environment with a 12 h light/dark cycle, and they received diet and water ad libitum. Experimental procedures on mice were approved by the Animal Care and Ethics Committee of the Institute of Radiation Medicine (approval number: 170619, 14 March 2017). Whole-body irradiation (WBI) and abdominal irradiation was performed using a ${}^{137}$Cs gamma-ray source (Gammacell-40, Atomic Energy of Canadian Inc., Chalk River, ON, Canada) at a dose-rate of 0.99 Gy/min. Control group was treated similarly to the irradiated groups but without exposure to ionizing radiation.

### 4.3. Survival Study

ICR mice were randomly divided into six groups (*n* = 24 each): control, vehicle + IR, 10 mg/kg 2-ADT + IR, 20 mg/kg 2-ADT + IR, 10 mg/kg 2-AADT + IR, 20 mg/kg 2-ADDT + IR. 2-ADT and 2-AADT were dissolved in normal saline for administration at the desired concentrations for intraperitoneal (i.p.) injection into the mice. For the 2-ADT and 2-AADT treatment , mice received an intraperitoneal injection of 0.2 mL solution once 30 min prior to 7.5 Gy WBI. the control and vehicle groups were treated with vehicle similarly to the procedure described above. Survival was observed daily up to 30 days post irradiation.

### 4.4. Determination of the Effect of 2-ADT and 2-AADT on Radiation Induced Hematopoietic System Injury

ICR mice were randomly divided into four groups (*n* = 8 each): control, vehicle + IR, 20 mg/kg 2-ADT + IR, 10 mg/kg 2-AADT + IR. Mice were treated as described above and exposed to 6.0 Gy irradiation. Mice were sacrificed by cervical dislocation 7 days post-irradiation. Blood, spleen, and bilateral femurs were collected to evaluate the effect of 2-ADT and 2-AADT on the hematopoietic system [15]. Blood was collected before cervical dislocation.

#### 4.4.1. Hematological Parameters

Blood samples were collected from the retro-orbital venous plexus into K${}_{2}$EDTA collection tubes for hematology analysis, and animals were anesthetized prior to retro orbital blood collections. The number of different types of blood cells in the peripheral blood was counted by a MEK-7222K hemocytometer (Nihon Kohden Corp, Tokyo, Japan).

#### 4.4.2. Colony Forming Assay

The spleens were removed and fixed in Bouins solution for 24 h. Macroscopic spleen colony-forming unit (CFU-S) on the splenic surfaces were scored [35].

#### 4.4.3. Bone Marrow Nucleated Cells and DNA Content

The bilateral femurs of the mice were excised and all muscle tissue was removed. For determination of bone marrow DNA content, the bone marrow was flushed into 10 mL 0.005 M calcium chloride solution. After placing the cell suspension 30 min at 4 ${}^{\circ }$C, centrifugation and discarding the supernatant, the sediment is resuspended in 5 mL of a 0.2 M perchloric acid. Suspension was incubated for 30 min at 90 ${}^{\circ }$C, filtered after cooling, and the absorbance was measured using an ultraviolet spectrophotometer at 268 nm [36]. For determination of bone marrow nucleated cells (BMNC), the bone marrow was flushed into 1ml PBS followed by filtered through nylon meshes for cytometric analysis [36].

### 4.5. Biochemical Determination

The livers and lungs were collected for biochemical determinations including MDA, SOD, GSH and CAT analysis using Malondialdehyde assay kit, Total Superoxide Dismutase assay kit, Reduced glutathione assay kit and Catalase assay kit (Nanjing Jiancheng Bioengineering Institute, Nanjing, China).

### 4.6. Plasma NOx Detection

ICR mice were randomly divided into four groups (*n* = 12 each): control, vehicle + IR, 20 mg/kg 2-ADT + IR, 10 mg/kg 2-AADT + IR. Mice were treated as described above in survival study. Blood samples were collected at 6 h and 7 days after 7.5 Gy WBI. Six mice were taken from each group at each time point. the concentration of plasma nitrite and nitrate (NOx) was measured using Nitric Oxide (NO) assay kit (Nitrate reductase method) (Nanjing Jiancheng Bioengineering Institute). Nitrite and nitrate (NOx) as stable metabolites of NO were used to quantify NO production [37].

### 4.7. Comet Assay

Whole blood was obtained from mice 1h after 6.0 Gy WBI. Peripheral blood lymphocytes were separated from the whole blood by using mouse peripheral lymphocyte separation medium (TBDsciences).the basic procedure is performed as described by Yang W. et al. [38]. 100 randomly selected cells each sample are analyzed and tail moment (TM) and percentage of DNA in tail (TDNA%) were selected for the quantification of DNA damage.

### 4.8. Determination of the Effects of 2-ADT and 2-AADT on Intestinal Injury in Irradiated Mice

C57BL/6 mice were randomly divided into four groups (*n* = 6 each): control, vehicle + IR, 20 mg/kg 2-ADT + IR, 10 mg/kg 2-AADT + IR. Mice were treated as described above, and anesthesia with an intraperitoneal injection of 3.5% chloral hydrate (0.1 mL/10g body weight) before 13 Gy abdominal radiation. the small intestine was collected at 3 days post irradiation and fixed in 4% paraformaldehyde for 24 h before paraffin embedding, and cut into 5 μm-thick sections.

#### 4.8.1. Histopathology

The sections were stained with hematoxylin and eosin (HE) and examined under a light microscope. Villus injury, crypt damage and mononuclear cell infiltration in the lamina propria were used to assess small intestine injury [39].

#### 4.8.2. Immunohistochemistry

The paraffin sections were deparaffinized and rehydrated using xylene and ethanol, and immersed in 3% hydrogen peroxide solution for 30 min to block endogenous peroxidase. the sections were immersed in Tris-EDTA Buffer (pH 9.0) by microwave heating for antigen retrieval. Sections were blocked with goat serum for 30 min and then incubated with anti-nitrotyrosine (1:200, ab42789) , anti-Ki67 (1:300, NB110-89717F), anti-lysozyme (1:800, ab108508), and anti-Villin (1:800, ab130751) antibodies at 4 ${}^{\circ }$C overnight in a humidified chamber. PBS-washed sections were then incubated with a horseradish peroxidase (HRP)-conjugated anti-rabbit detection system (PV-6001, ZSGB-BIO, Beijing, China) for 1h at room temperature. After washing, the slides were visualized with DAB and counterstained with hematoxylin. For each mouse, four cross-sections of the intestine were cut and four views were captured randomly in each cross-section using a microscope. the images were quantified by measuring the density and number of DAB-stained cells with Image Pro Plus 6.0 software (Media Cybernetics, Bethesda, MD, USA) [40].

### 4.9. Statistical Analysis

The results were presented as mean ± SD. Statistical analysis was performed by one-way ANOVA using SPSS 19.0 (SPSS Inc, Chicago, IL, USA). Statistical significance was taken as *p* < 0.05 or *p* < 0.01. the significance of survival curves was analyzed by Kaplan–Meier survival analysis and the log-rank test.
